# Perspectives of Historically Black College and University Advisors to Premedical Students During the COVID-19 Pandemic: A Qualitative Study

**DOI:** 10.1001/jamanetworkopen.2022.38563

**Published:** 2022-10-21

**Authors:** Jasmine Weiss, Louisa Holaday, Danya Keene, Ngozi D. Akingbesote, Lilanthi Balasuriya, Mona Sharifi, Darin Latimore, Inginia Genao

**Affiliations:** 1 Division of General Pediatrics and Adolescent Medicine, Department of Pediatrics, University of North Carolina School of Medicine, Chapel Hill; 2 Division of General Internal Medicine, Department of Medicine, Icahn School of Medicine at Mount Sinai, New York, New York; 3 Institute for Health Equity Research, Icahn School of Medicine at Mount Sinai, New York, New York; 4 Department of Social and Behavioral Sciences, Yale School of Public Health, New Haven, Connecticut; 5 Department of Cellular and Molecular Physiology, Yale University School of Medicine, New Haven, Connecticut; 6 Department of Internal Medicine (Endocrinology), Yale University School of Medicine, New Haven, Connecticut; 7 Yale National Clinician Scholars Program, Yale University School of Medicine, New Haven, Connecticut; 8 Department of Pediatrics, Yale University School of Medicine, New Haven, Connecticut; 9 Department of Internal Medicine, Yale University School of Medicine, New Haven, Connecticut; 10 Department of Medicine, Penn State College of Medicine, Hershey, Pennsylvania

## Abstract

**Importance:**

Black students remain underrepresented in medicine despite national efforts to increase diversity in the physician workforce. Historically Black College and University (HBCU) students play a vital role in increasing representation in the workforce. Currently, there is a paucity of literature understanding the impact of COVID-19 on premedical students from HBCUs. Understanding the adverse impact of the pandemic on HBCU students is essential to inform strategies that promote holistic medical school admissions and increased diversity, equity, and inclusion in the medical workforce.

**Objective:**

To explore premedical advisors' perspectives on the impact of the COVID-19 pandemic on HBCU premedical students pursuing admission to medical school.

**Design, Setting, and Participants:**

In this qualitative study, semistructured interviews of HBCU premedical advisors were performed from March 2020 to March 2021. One-on-one interviews were conducted with 21 advisors with a depth of experience as advisors, varied educational backgrounds, and diverse geographic representation. Data analysis was performed from March 2021 to December 2021.

**Main Outcomes and Measures:**

The experiences of HBCU premedical students during the COVID-19 pandemic from the perspective of the premedical advisor.

**Results:**

Among the 21 participants, 13 (62%) were female, 15 (71%) were Black or African American, 11 (52%) had a doctorate degree, and 7 (33%) had more than 10 years of experience as advisors. Participants described 3 major themes: (1) balancing academic responsibilities with family demands; (2) distraction, disruption, and isolation in the virtual learning environment; and (3) harmful impact of new stressors for HBCU applicants in the medical school admissions process.

**Conclusions and Relevance:**

In this qualitative study of HBCU advisors to premedical students, advisors described how the COVID-19 pandemic adversely affected undergraduate HBCU premedical students; students faced family hardships, challenges with virtual learning, and uncertainty in the medical school admissions process. These findings suggest that medical schools should continue to create direct interventions to address the challenges that HBCU students faced during the height of the pandemic and as longitudinal consequences of the pandemic. Addressing these issues may improve physician workforce representation and promote more equitable patient care for underserved communities disproportionately affected by COVID-19 and other health disparities.

## Key Points

**Question:**

How do premedical student advisors describe the impact of COVID-19 on premedical students at historically Black colleges and universities (HBCUs) pursuing admission to medical school?

**Findings:**

This qualitative study of interviews with 21 premedical advisors from HBCUs described 3 major ways that the pandemic affected premedical students: balancing academic responsibilities with family demands, difficulty with the virtual learning environment, and new stressors for medical school applicants.

**Meaning:**

These findings suggest that the pandemic adversely affected HBCU premedical students, which has implications for the well-being of individual students, their medical school preparation, and physician workforce diversity.

## Teaser

This qualitative study reports premedical student advisor perspectives on how the COVID-19 pandemic affected historically Black college and university (HBCU) premedical students pursuing admission to medical school.

## Introduction

Health disparities adversely affect the Black community with poor outcomes for major health indicators such as infant mortality, maternal mortality, and life expectancy.^1,2,3^ The COVID-19 pandemic further exacerbates health inequities with disparities in morbidity and mortality in urban and rural areas, and in counties with a higher proportion of Black individuals throughout the United States.^4,5,6,7,8,9,10^ A contributing factor to worse health outcomes is the lack of racial and ethnic representation in the physician workforce, particularly among Black physicians.

Given these existing inequalities, it is important to understand how the COVID-19 pandemic may affect representation in the physician workforce through its impacts on medical education. While some research has documented impact of pandemic on medical education at all levels, the impact on premedical education has not been fully characterized for Black students. Globally, undergraduate students have experienced increased stress, anxiety, and depression since the onset of the pandemic.^11,12,13,14^ Undergraduate students were required to transition to remote learning, and some were required to relocate from university campuses abruptly at the start of the pandemic.^15^ One setting to further explore the impact of COVID-19 on Black premedical students is to characterize the experiences of Black students at Historically Black Colleges and Universities (HBCUs). HBCUs make up 3% of all colleges and universities in the US yet account for 24% of all African American graduates earning baccalaureate degrees nationwide, and 25% of all African Americans graduates earning STEM (science, technology, engineering, and math) degrees.^16,17^

Our study evaluates the impact of COVID-19 on HBCU premedical students pursuing admission to medical school from the perspective of the premedical advisor. In this qualitative study, we conducted one-on-one in-depth interviews with HBCU premedical advisors to identify themes related to the experiences of premedical students since the outset of the COVID-19 pandemic. This has important implications for future representation in the health care workforce and the care of individuals from historically underserved communities.

## Methods

### Study Sample

The participants recruited for this study were initially identified as part of a larger qualitative study to assess current practices of premedical advisors at HBCUs. A premedical advisor was defined as a designated individual at a university who provided academic advising to students pursuing admission to medical school. We recruited the advisors using purposive sampling and identified study participants based on their knowledge and interactions with undergraduate students interested in pursuing medical school.^18^ Participants were identified at the Yale School of Medicine 2019 First Look Immersion sponsored by the Office of Diversity and Inclusion Community Engagement and Equity. During the event, students, and premedical advisors from HBCUs were invited to Yale School of Medicine to learn more about the medical school and its admissions process. We conducted 1-on-1 interviews (n = 21) via zoom from March 2020 to March 2021.The analysis in this paper draws on these interviews to explore the impact of COVID-19 on HBCU students interested in medical school through the lens of the premedical advisor. Participants provided verbal informed consent, including permission to publish deidentified quotes. The Yale institutional review board approved this study. We reported our study results using the Consolidated Criteria for Reporting Qualitative Research (COREQ) reporting guideline for qualitative research.^19^

### Data Collection

An author (J.W.) conducted semistructured interviews using an interview guide with open-ended questions to assess standard practices for advising students and any challenges they observed as students matriculated through the admissions process. With the onset of COVID-19, questions were added in an iterative manner to capture advisors’ perceptions of how the pandemic impacted premedical students (Box). We initially pilot tested the interview guide with 2 health services researchers (J.W. and L.H.). We iteratively revised the interview guide based on participant responses in subsequent interviews.

Box. **COVID-19 Interview Questions**

1. Can you describe how COVID 19 has impacted your premedical students?
2. Can you share how the pandemic has had a negative impact on your students?
  - Positive impact?
3. Can you describe the medical school’s responses to the pandemic?
4. Can you describe student perceptions of changes to the admissions process?
5. Can you describe any specific changes to curriculum due to the pandemic?

During the interview, the discussion allowed for deviation from the guide based on participant responses. Probing questions were asked when additional themes were raised. Interviews were audio recorded and transcribed verbatim. We interviewed participants until thematic saturation was reached, defined as the point at which no new concepts emerged during interviews.^20^

Data regarding the participant’s duration in their role as a premedical advisor and demographic characteristics were also collected via survey. Demographic data included self-reported race and ethnicity (Asian, Black or African American, Hispanic/Latinx, White), sex (female, male), age (aged 25 to 34 years, 35 to 44 years, 45 to 54 years, and at least 55 years). Participants who did not self-identify with any of the subcategories of race and ethnicity provided were given the option to choose other. This option was not chosen by any of the participants.

### Data Analysis

The study team analyzed the data using thematic analysis, in which data were coded line-by-line and subsequently organized into themes. Codes were developed based on participant responses that were similar in nature. The initial coding was used to establish a finalized codebook that was then applied to all transcripts (J.W. and L.B.). We coded the first 5 transcripts together to ensure interrater reliability then subsequently coded the data individually and met regularly to discuss data interpretation (J.W. and N.A.). Similar codes were categorized into themes. We used NVivo software version 12 (QSR International) for data storage and organization.

## Results

### Participant Characteristics

Participants included 21 premedical advisors from HBCUs with varying levels of advising experience, representing a wide array of geographic locations and educational levels. Some premedical advisors also served in dual capacities such as course instructors for students. Among the 21 participants, 15 (71%) self-identified as Black and 13 (62%) as female. The Table displays the characteristics of study participants.

**Table.** Demographics of Participating HBCU Premedical Advisors

Characteristics	Participants, No (%)^a^
Sex	
Male	8 (38)
Female	13 (62)
Age, y	
25-34	1 (5)
35-44	6 (28)
45-54	5 (24)
≥55	9 (43)
Race or ethnicity	
Asian	3 (14)
Black or African American	15 (71)
Hispanic/Latinx	1 (5)
White	2 (10)
Education level	
Master’s degree (ie, MA, MS, Med)	6 (29)
Professional degree (ie, MD, DDA, DVM)	4 (19)
Doctorate (ie, MD, PhD, EdD)	11 (52)
Formal training for advising premedical students	
Yes	3 (14)
No	18 (86)
Experience as premedical advisor	
<1 y	1 (5)
1-2 y	2 (10)
2-5 y	6 (28)
6-10 y	5 (24)
>10 y	7 (33)
Region	
North region	
Mid-Atlantic division	2 (10)
South region	
South Atlantic division	12 (57)
East South-Central division	7 (33)

Abbreviation: HBCU, historically Black colleges and universities.

^a^ Percentages may not sum to 100 owing to rounding.

### Themes

Premedical advisors described the following 3 major themes concerning their perception of the difficulties that students faced during COVID-19: (1) balancing academic responsibilities with family demands; (2) distraction, disruption, and isolation in the virtual learning environment; and (3) harmful impact of new stressors on HBCU applicants in the medical school admissions process.

#### 1. Balancing Academic Responsibilities With Family Demands

##### Coping With Illness or Death of Family Members

Advisors reported that their students had to contend with difficult family circumstances related to the pandemic. Many students had to cope with family illness and loss, while simultaneously shouldering their academic responsibilities. As one advisor noted, “In spring, I had quite a few students who had grandparents that died in my classes, in my lab. There almost wasn’t one student that I can remember who was untouched by someone in their family. Most of the students…knew someone related to them who had gotten coronavirus, and many of them knew people who had died.” Most participants regarded these circumstances as detrimental to the students’ ability to focus on their studies. Dealing with the death and illness of family members was only one challenge that students faced when returning home during the pandemic.

##### Supporting Family Economically

Many advisors also noted that students had to balance academic responsibilities with financial needs of their family members. With many students’ family members working in low-paying unstable jobs during the pandemic, students often stepped in to ease their family’s economic burden. As one participant noted, “I had a student…in my class this semester. [An] African American male doing excellent in my class. He was in a blended synchronous learning class. After midterms, he dropped the class, and I asked him, ‘What happened?’ He said, ‘My job. I had to do so much.’ And he was doing so well. But the priority is you got to feed your family, right?” Many advisors highlighted that students’ felt obligated to seek employment to assist their families given the economic strain imposed by the pandemic.

#### 2. Distraction, Disruption, and Isolation in the Virtual Learning Environment

##### Family Distraction and Limited Technology in the Virtual Learning Environment

While navigating challenges to their family’s health and economic status, the shutdown of campus facilities required students to return home and continue their studies in close physical proximity of their family. Many premedical advisors noted families often had difficulty understanding the needs of their college student and the time commitment needed to focus academically. One participant noted, “You take a student that is used to campus [life]… You throw them back at home, and momma expects them to clean their room and help with this, and “Can you stay with so-and-so while I go grocery shopping”. All these things are on top of everything else they are doing. Families don’t necessarily have the same ability to understand what the student is going for.”

Many participants reported cases of students being challenged by the lack of access to technology. There was also a need to share resources among family members for college students in the virtual setting. “Some families only have 1 or 2 computers so they have limited broadband so if there’s younger children at home and they have to do school then their older siblings may not have access to the computer during school hours.” Advisors noted that physical environment constraints, lack of technology resources, and family distractions all contributed to poor academic performance for some students. “I have students who were 3.6, 3.8, 3.9 GPA. This hit and they barely made it through their classes.” In the typical setting an advisor could provide a struggling student with academic support, but because of the pandemic these interactions were limited.

##### Lack of Advisor-Student Interactions

Prior to the pandemic, premedical advisors described not only providing academic support, but also in-person emotional support for students. They reported serving as confidants for a student struggling with family challenges or monetary obstacles, however, the pandemic strained their ability to interact with students. One participant stated, “So all of these support systems … were a little shaky because we used to be able to approach our students. If they need a hug, we'd give them a hug. It’s that personal touch. It’s very difficult to do that across a camera.”

Participants reported that the lack of physical interaction also limited the advisor’s ability to engage with newer students to help them acclimate to college life. “Because we haven't actually gotten a chance to meet this cohort that we have taken in, there's a feeling of disconnectedness … we are going to have to beef up our game. It’s very difficult to establish a relationship virtually with faces on a screen.” Advisors highlighted that fewer students participated in virtual advising opportunities when compared with in-person opportunities in the previous years.

##### Isolation From Peers

Premedical advisors reported that the perception of disconnectedness not only applied to relationships between advisors and students, but it also interfered with student interactions with one another. One participant noted, “They're isolated…only in the last month, I invited all of them to my house, very first time, very first social thing in a whole year… they didn't even know each other. They maybe saw each other some time, but they were introducing themselves for the first time. Now, imagine if we could have done that earlier in the year. We would have built a community much sooner. Here we are, they barely know each other…I think it’s affected them a lot of ways.” Participants reported that the lack of advisor-student interaction and peer support in the virtual learning environment also affected the ability of students to navigate the medical school admissions process.

#### 3. Harmful Impact of COVID-19 Stressors on HBCU Applicants in the Medical School Admissions Process

##### Constrained Clinical Shadowing Opportunities

Participants highlighted a decrease in the students’ ability to obtain in-person clinical shadowing experiences due to the pandemic. “All of a sudden, students are not able to interact, get into the community, go to the hospitals, go to nursing homes, assisted living facilities, and shadow physicians…opportunities that they’ve always been a part of, all of a sudden, they are not able to be a part of those opportunities and that also created some stress and some strain on a number of our students.”

Advisors also expressed concern for students with limited prior clinical experiences. “A lot of students start off without any exposure [to clinical experiences] when they come to campus. So, they have a very short period to gain…that exposure that they need…to be competitive on an application. This just threw a wrench into it.” Advisors noted frequent communication with medical schools to promote virtual shadowing opportunities to mitigate the lack of in-person opportunities.

##### Recurring Shifts in the Medical College Admission Test Schedule

Similarly, students had to navigate recurring changes to the Medical College Admission Test (MCAT) testing schedule while preparing to take the exam. “These … students [are] trying to take the MCAT. Well, all of a sudden, their test date’s getting pushed back and when they have to go home and the university is closed, students have to take care of their loved ones. Some students are living with … extended families where you have 10, 12 people in the home, and it made it impossible for students to be able to… have that quiet time to study.”

Advisors also reported that the stress of the admissions process during the pandemic negatively impacted the well-being of the students. “Each time [the MCAT] gets pushed back, they fall into this mode whereas they have to try to go back and…study more and it’s just it really has created a situation for students where some students even had to go to counseling because they were so stressed out [with] higher levels of anxiety.”

##### Uncertainty With Changes to Medical Schools’ Admission Criteria

Advisors were also unclear on how institutions would consider shadowing opportunities, test scores, and academic performance during the pandemic. There was variability in messaging regarding how the pandemic would affect the admissions process. Some received information that schools were going to be flexible; other participants were concerned that schools would not change the process. One participant stated, “From what I've heard, the medical schools aren't letting up on admissions criteria. So, it’s still GPA, MCAT score. And you get in or you don’t.”

With uncertainty regarding how the admissions process would change, some advisors were concerned that students with fewer resources would be negatively impacted by these changes and their circumstances. “Maybe there’s a student that had a 3.9 and just didn’t score well on the MCAT, or maybe had some really hard things going on. So how do you balance that compared to a student especially from a non–minority group who may have more resources, more money, more access to test prep and things like that.”

## Discussion

In this qualitative study, many premedical advisors described perceptions that HBCU students interested in medical school were negatively impacted by the COVID-19 pandemic. For the premedical advisors charged with the task of providing students guidance into the medical school admissions process, advisors described how the pandemic changed both their advising strategies and student life overall. Study participants described student challenges with balancing academic responsibilities and family needs, difficulty with the virtual learning environment, and new stressors causing setbacks for applicants in the medical school admissions process. These challenges are likely to have implications for student well-being, for navigating the medical school admissions process, and for future representation of Black trainees in the physician workforce.

Advisors noted, across multiple themes, that multifactorial stressors related to the pandemic impacted student well-being. For college students globally, the pandemic has contributed to worsening stress, anxiety, and depression.^12,13,21^ The present study highlights how these issues specifically impact premedical students at HBCUs in the context of dealing with family illness, variable living situations, and economic hardships. The racial disparities in morbidity and mortality due to COVID-19 in the African American community may exacerbate these issues for HBCU premedical students who are predominantly from this community.^10,22,23,24^ Advisors, faculty, and institutions at large must consider additional interventions to ensure that the mental health needs of students are addressed, and resources are available to support students’ overall well-being during this and similar crises.

For historically underrepresented students navigating the admission process, evidence suggests that supportive peer networks, relationships with mentors, and research opportunities promote retention and the pursuit of health careers.^25,26,27,28^ Yet, prior to the pandemic, students at HBCUs perceived barriers such as a lack of mentorship opportunities, institutional support, and shadowing opportunities.^29^ Our study illuminates the virtual learning environment exacerbated the scarcity of these resources for HBCU students. This may have downstream effects on the medical school admissions process for HBCU students in the future.

Our findings highlight challenges related to medical school admissions, including the MCAT. This is a known barrier to medical school admission for historically underrepresented students.^30^ In the context of the pandemic, students have endured MCAT study disruptions, delays in MCAT testing, requirements for social distancing, and the use of PPE during testing.^31,32^ Furthermore, students had to make the abrupt transitions to virtual interviews for medical school.^33,34^ For HBCU students predominantly from communities disproportionality impacted by COVID-19, there were additional responsibilities including taking care of family members, seeking employment to support their families, and challenges with access to technology in their home environment. Participants described cases in which these responsibilities translated into less flexibility and fewer financial resources for MCAT preparation.

Despite formalized adjustments to the Medical School Admissions Requirements by the Association of American Medical Colleges, participants described concern for variability in the uptake of recommendations by individual schools. Some advisors raised concern that individualized challenges associated with the pandemic may be overlooked in evaluating a student’s medical school application. These concerns support the need for medical schools to continue to use a holistic review process to ensure that academic and nonacademic experiences are strongly considered along with standardized test scores.^35,36,37^ Medical schools should be proactive in considering the effects of the pandemic for HBCU students during current and future admission cycles. This is especially critical in the setting of a projected shortage of 122 000 physicians by the year 2032.^38,39,40,41,42^

### Limitations

One limitation of this study is that participants in our study were mostly Black advisors from HBCUs, thus our data may not capture the full experience of Black students who attend undergraduate institutions that are not designated as HBCUs. Although this study uses qualitative methodology, it is important to consider quantitative methods and large-scale studies with historically underrepresented premedical students for further generalizability.

## Conclusions

In this qualitative study, our findings demonstrated how the COVID-19 pandemic adversely affected HBCU premedical students through family hardships, economic obstacles, difficulty with the virtual learning environment, and constrained educational opportunities. This has implications for the well-being of individual students, medical school preparation, and workforce diversity at large. These findings elucidate the need for increased resources, mentorship, and premedical opportunities for HBCU students post pandemic. Longitudinal studies are needed to assess the impact of the pandemic on students throughout their medical training. This is critical to ensuring a sustained increase in workforce representation that contributes to more equitable patient care for underserved communities disproportionately affected by COVID-19 and other health disparities.
